# Baseline CALLY index is associated with all-cause mortality in HBV-ACLF patients receiving ALSS therapy

**DOI:** 10.3389/fmed.2026.1827310

**Published:** 2026-08-21

**Authors:** Wanlong Ma, Jinhua Yuan, QiuYan Chen, Xia Wang, Le Zhang, Yanghua Ou, Yun Jiao, XiangChun Ding

**Affiliations:** 1 Department of Infectious Disease, General Hospital of Ningxia Medical University, Yinchuan, Ningxia, China; 2 Ningxia Medical University, Yinchuan, Ningxia, China; 3 Infectious Disease Clinical Research Center of Ningxia, Yinchuan, Ningxia, China

**Keywords:** acute-on-chronic liver failure, artificial liver support system, CALLY index, HBV, mortality

## Abstract

**Objective:**

Chronic hepatitis B virus (HBV)-associated acute-on-chronic liver failure (HBV-ACLF) has a high mortality rate even with the use of artificial liver support systems (ALSS). Reliable prognostic biomarkers are lacking. This study investigates the association between the C-reactive protein (CRP)-albumin-lymphocyte (CALLY) index, measured at diagnosis, and all-cause mortality in HBV-ACLF patients receiving ALSS treatment.

**Methods:**

This single-center retrospective cohort study enrolled consecutive adult patients with HBV-ACLF who received ALSS therapy at the General Hospital of Ningxia Medical University between May 2022 and November 2025. Baseline CALLY was calculated before ALSS initiation. Restricted cubic spline analysis, Kaplan–Meier survival analysis, and Cox regression were used to evaluate the association between CALLY and all-cause mortality. Logistic regression was used to assess its associations with major complications. Time-dependent ROC curves, Brier scores, calibration curves, and decision curve analysis were applied to evaluate the predictive performance and clinical utility of CALLY alone and in combination with conventional prognostic scores.

**Results:**

A total of 221 patients with HBV-ACLF were included, among whom 99 experienced all-cause mortality during follow-up. Patients with low CALLY had more severe systemic inflammation, poorer nutritional and immune status, higher proportions of hepatic encephalopathy and renal insufficiency, and more advanced disease severity than those with high CALLY. Restricted cubic spline analysis showed a significant nonlinear inverse association between baseline CALLY and all-cause mortality risk. Kaplan–Meier analysis demonstrated significantly better overall survival in the high-CALLY group than in the low-CALLY group (log-rank *P* < 0.0001). In Cox regression analysis, higher CALLY was consistently associated with a lower risk of all-cause mortality. Time-dependent ROC analysis showed that CALLY had good discriminative ability for predicting all-cause mortality at 3, 6, and 12 months, with AUCs of 0.822, 0.837, and 0.888, respectively. The addition of CALLY to AARC grade, Child–Pugh score, MELD score, and MELD-Na score significantly improved the AUCs of the corresponding models. Brier score, calibration curve, and decision curve analyses further supported the incremental predictive value and clinical utility of CALLY?

**Conclusion:**

The baseline CALLY index was inversely associated with all-cause mortality in HBV-ACLF patients receiving ALSS therapy and may enhance prognostic assessment, especially when integrated with conventional prognostic scoring systems.

## Introduction

Acute-on-chronic liver failure (ACLF) is a syndrome characterized by rapid progression, high short-term mortality, and particularly poor outcomes in the absence of liver transplantation. Hepatitis B virus (HBV)-related liver disease represents one of its major etiologies (1, 2). Although definitions differ across international consortia, ACLF is generally defined as acute decompensation in patients with underlying chronic liver disease, accompanied by systemic inflammation, immune dysregulation, and extrahepatic organ failure (3, 4). Hepatic encephalopathy and renal dysfunction are among the most frequent complications, and renal failure is strongly associated with an increased risk of death (5).

Artificial liver support systems (ALSS) can alleviate hepatic burden, facilitate hepatocyte regeneration, and increase the opportunity for liver transplantation; in some patients, ALSS may also serve as an adjunct to standard medical therapy and potentially obviate the need for transplantation (6, 7). In China, the most widely used ALSS modalities include plasma exchange (PE) and the double plasma molecular adsorption system (DPMAS) (6, 7). However, the clinical benefit of ALSS is highly heterogeneous, underscoring the urgent need for robust and readily available markers to enable rapid risk stratification and guide individualized treatment strategies and resource allocation.

Conventional prognostic models, including the Child-Pugh (CTP), model for end-stage liver disease (MELD), and MELD-Na scores, have limited predictive performance because they insufficiently capture extrahepatic organ failure and systemic inflammation, while the CTP score also incorporates subjective variables (8). Although the Lille score, CLIF-C ACLF, CLIF-SOFA, and the APASL ACLF Research Consortium (AARC) grade have been used to assess disease severity and prognosis, their calculation is relatively complex, and some components are susceptible to therapeutic interventions and dynamic clinical fluctuations. Moreover, their accuracy, cost-effectiveness, and generalizability across different etiologies—particularly in HBV-related ACLF—remain to be fully established, and evidence supporting their use for individualized prognostic assessment following ALSS is still limited (4).

Systemic inflammation is a central pathophysiological hallmark of ACLF, often coexisting with immune paralysis that predisposes patients to secondary infections. In parallel, inflammation-driven metabolic reprogramming and nutrient redistribution may further exacerbate organ injury (9–11). The CALLY index may provide an integrated reflection of inflammatory, nutritional, and immune status, and thus may represent a simple, inexpensive, and reproducible tool for risk stratification. Therefore, this study aimed to investigate the prognostic value of the baseline CALLY index for predicting all-cause mortality in patients with HBV-related ACLF undergoing ALSS, with the goal of informing pre-treatment risk assessment and clinical decision-making.

## Methods

### Study design and participants

This was a single-center retrospective cohort study. We consecutively enrolled adult inpatients (aged ≥18 years) who were newly diagnosed with hepatitis B virus-related acute-on-chronic liver failure (HBV-ACLF) and received artificial liver support system (ALSS) treatment at the General Hospital of Ningxia Medical University between May 1, 2022 and November 1, 2025.

HBV-ACLF was diagnosed according to the criteria of the Asian Pacific Association for the Study of the Liver (APASL), defined as hepatitis B surface antigen (HBsAg) and/or hepatitis B virus DNA (HBV DNA) positivity for at least 6 months, with jaundice [total bilirubin (TBIL) ≥5 mg/dl (≥85 μmol/L)] and coagulopathy [international normalized ratio (INR) ≥1.5 or prothrombin activity < 40%], complicated within 4 weeks by the development of ascites and/or hepatic encephalopathy (HE) (3, 4).

**Figure 1:**
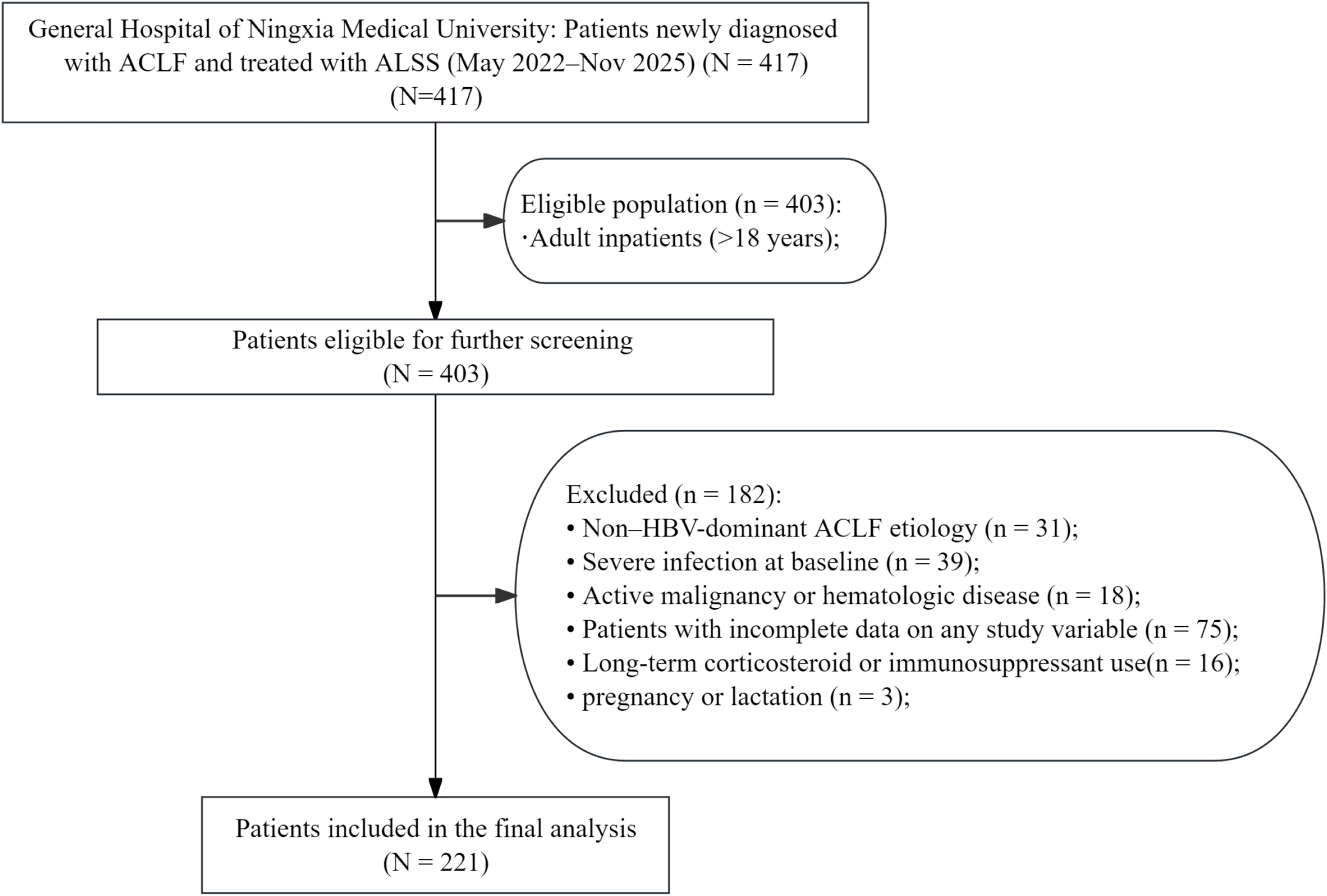
Flowchart of patient selection. A total of 221 patients were included in the final analysis. During follow-up, 1 patient underwent liver transplantation, 99 patients died, and 121 patients were censored.

The exclusion criteria were as follows: (1) ACLF predominantly caused by etiologies other than HBV; (2) documented severe infection at baseline; (3) active malignancy or hematologic disease; (4) patients with incomplete data on any study variable were excluded; (5) long-term use of glucocorticoids or immunosuppressants; and (6) pregnancy or lactation. The patient selection process is shown in Figure 1.

This study was approved by the Ethics Committee of the General Hospital of Ningxia Medical University (Approval No. KYLL-2023-0235).

### Data collection and definitions

Baseline data were collected at initial diagnosis and before the first ALSS treatment. Demographic and clinical variables included age, sex, and histories of hypertension, diabetes mellitus, and cardiovascular disease. Laboratory parameters included white blood cell count, neutrophil count, lymphocyte count, hemoglobin, platelet count, alanine aminotransferase (ALT), aspartate aminotransferase (AST), total bilirubin, albumin, international normalized ratio (INR), C-reactive protein (CRP), interleukin-6 (IL-6), and estimated glomerular filtration rate (eGFR). Conventional prognostic scores, including AARC grade, Child–Pugh score, MELD score, and MELD-Na score, were also recorded. ALSS-related information, including treatment modality and number of treatment sessions, was obtained from medical records.

Baseline complications included hepatic encephalopathy (HE) and renal insufficiency. HE was identified according to the West Haven criteria or explicit documentation in the medical records. Renal insufficiency was defined as eGFR < 60 ml/min/1.73 m^2^. The CALLY index was calculated as follows: CALLY = albumin (g/L) × lymphocyte count ( × 10^9^/L) / CRP (mg/dl).

### Treatment protocol

All patients received standard comprehensive medical treatment in accordance with routine clinical practice, including supportive care, antiviral therapy when indicated, hepatoprotective therapy, correction of metabolic disturbances, supplementation with albumin and/or blood products, and prevention and management of complications.

Baseline blood samples were collected 1 day before the first ALSS session. ALSS was initiated after the attending physicians had comprehensively evaluated disease severity, coagulation status, and hemodynamic stability. The ALSS modalities included the double plasma molecular adsorption system (DPMAS) alone or DPMAS combined with plasma exchange (PE). The choice of treatment modality was individualized according to each patient's clinical condition.

Repeated ALSS sessions were considered in patients with persistent or worsening hyperbilirubinemia, coagulopathy, hepatic encephalopathy, renal dysfunction, systemic inflammation, or an inadequate clinical response to the preceding session, provided that they remained hemodynamically stable and able to tolerate further treatment. The specific ALSS modality and total number of sessions were determined according to the patient's clinical response and the consensus assessment of at least three senior physicians.

### Outcomes

The primary outcome was all-cause mortality. The last follow-up date was November 22, 2025. Overall survival (OS) was defined as the interval from the date of blood sample collection performed 1 day before the initial ALSS treatment to the date of death or last follow-up.

### Statistical analysis

All statistical analyses were performed using R software (version 4.4.3). Continuous variables were expressed as mean ± standard deviation (SD) or median with interquartile range (IQR), as appropriate, and were compared using the two-sample *t*-test or the Mann–Whitney *U*–test. Categorical variables were presented as *n* (%) and compared using the χ^2^ test or Fisher's exact test. All analyses were performed based on the complete-case dataset.

Patients were categorized into low- and high-CALLY groups according to the median baseline CALLY value. Restricted cubic spline analysis was used to explore the dose–response relationship between baseline CALLY and the risk of all-cause mortality, with the median CALLY value used as the reference point. The analysis was restricted to the 5th−95th percentile range. Kaplan–Meier survival curves were generated according to CALLY groups and compared using the log-rank test.

Cox proportional hazards regression models were used to evaluate the association between CALLY and all-cause mortality. CALLY was analyzed as a continuous variable, a log2-transformed variable, a binary variable based on the median value, and a tertile-based categorical variable. The crude model was unadjusted; Model 1 was adjusted for age and sex; and Model 2 was further adjusted for histories of hypertension, diabetes mellitus, and cardiovascular disease. A sensitivity analysis was performed by censoring the patient who underwent liver transplantation at the time of transplantation. Logistic regression models were used as supplementary analyses to assess the cross-sectional associations between baseline CALLY and major complications.

Time-dependent receiver operating characteristic (ROC) curves were constructed at 3, 6, and 12 months to evaluate the discriminative performance of CALLY, conventional prognostic scores, and CALLY-based combined models for predicting all-cause mortality. Brier scores were calculated to assess overall prediction error. Calibration curves were used to evaluate the agreement between predicted and observed mortality risks, and decision curve analysis was performed to assess clinical net benefit across different threshold probabilities.

## Results

### Baseline clinical characteristics

A total of 221 patients with HBV-ACLF were included in the analysis, of whom 99 experienced all-cause mortality during follow-up and 122 were classified into the non-death group. Compared with survivors, patients in the death group were older and had higher rates of hepatic encephalopathy and renal failure (all *P* < 0.05). They also had lower lymphocyte counts, hemoglobin, platelet counts, ALT, AST, and albumin levels, but higher INR, CRP, IL-6, Child–Pugh scores, MELD-Na scores, and AARC grades (all *P* < 0.05). No significant differences were observed in sex, comorbidities, white blood cell count, neutrophil count, total bilirubin, MELD score, ALSS modality, or number of ALSS sessions (Table 1).

**Table 1:** Baseline characteristics according to mortality event status during follow-up.

Characteristics	Overall (*N* = 221)	Death group (*N* = 99)	Non-death group (*N* = 122)	*P*
Demographic characteristics				
Age (years), mean ± SD	48.93 ± 11.15	52.41 ± 10.51	46.10 ± 10.89	< 0.001
Sex, *n* (%)				0.284
Female	57 (25.79)	29 (29.29)	28 (22.95)	
Male	164 (74.21)	70 (70.71)	94 (77.05)	
Hypertension history (Yes), *n* (%)	15 (6.79)	10 (10.10)	5 (4.10)	0.078
Diabetes history (Yes), *n* (%)	18 (8.14)	7 (7.07)	11 (9.02)	0.599
Cardiovascular disease history (Yes), *n* (%)	10 (4.52)	4 (4.04)	6 (4.92)	>0.999
Baseline complications				
Hepatic encephalopathy (Yes), *n* (%)	51 (23.08)	32 (32.32)	19 (15.57)	0.003
Renal insufficiency (Yes), *n* (%)	15 (6.79)	13 (13.13)	2 (1.64)	< 0.001
Baseline laboratory parameters and scoring systems				
White blood cell count ( × 10^9^/L), median (IQR)	5.95 (4.20, 8.19)	5.48 (4.10, 8.28)	6.22 (4.40, 8.12)	0.468
Neutrophil count ( × 10^9^/L), median (IQR)	3.66 (2.53, 5.39)	3.47 (2.52, 5.69)	3.88 (2.58, 4.98)	0.864
Lymphocyte count ( × 10^9^/L), median (IQR)	1.08 (0.80, 1.54)	0.77 (0.51, 1.00)	1.44 (1.08, 1.84)	< 0.001
Hemoglobin (g/L), median (IQR)	126.00 (108.00, 146.00)	117.00 (89.00, 136.00)	135.50 (119.00, 148.75)	< 0.001
Platelet count ( × 10^9^/L), median (IQR)	105.00 (72.00, 152.00)	82.00 (50.00, 126.50)	121.50 (89.25, 176.50)	< 0.001
ALT (U/L), median (IQR)	175.20 (67.30, 504.70)	97.30 (43.35, 354.10)	295.70 (121.63, 615.32)	< 0.001
AST (U/L), median (IQR)	197.60 (105.90, 421.80)	140.10 (82.90, 349.05)	255.55 (147.10, 543.38)	< 0.001
Total bilirubin (μmol/L), median (IQR)	308.50 (228.47, 388.00)	322.40 (232.90, 430.20)	295.55 (226.25, 374.05)	0.055
Albumin (g/L), median (IQR)	30.70 (27.85, 34.30)	29.60 (26.52, 32.55)	31.99 (29.02, 35.40)	< 0.001
INR, median (IQR)	1.77 (1.51, 2.16)	1.78 (1.61, 2.33)	1.69 (1.44, 2.08)	0.006
CRP (mg/L), median (IQR)	10.50 (6.42, 19.60)	17.80 (10.70, 35.55)	7.73 (4.99, 11.78)	< 0.001
IL-6 (pg/ml), median (IQR)	11.40 (5.54, 27.30)	22.40 (13.45, 59.70)	6.90 (4.04, 12.42)	< 0.001
AARC grade, *n* (%)				< 0.001
I	90 (40.72)	15 (15.15)	75 (61.48)	
II	101 (45.70)	59 (59.60)	42 (34.43)	
III	30 (13.57)	25 (25.25)	5 (4.10)	
Child-Pugh score, median (IQR)	11.00 (10.00, 12.00)	12.00 (11.00, 13.00)	10.00 (9.00, 12.00)	< 0.001
MELD score, median (IQR)	19.19 (16.31, 22.06)	19.64 (15.92, 24.31)	19.05 (16.36, 21.11)	0.099
MELD-Na score, median (IQR)	22.73 (19.19, 32.33)	29.37 (23.96, 40.00)	20.01 (18.28, 23.34)	< 0.001
ALSS modality, *n* (%)				0.356
DPMAS	20 (9.05)	7 (7.07)	13 (10.66)	
DPMAS + PE	201 (90.95)	92 (92.93)	109 (89.34)	
Number of ALSS sessions, median (IQR)	3.00 (2.00, 4.00)	3.00 (2.00, 4.00)	3.00 (2.00, 4.00)	0.393
Follow-up time, (months) median (IQR)	7.00 (2.30, 18.40)	2.40 (1.35, 3.85)	17.95 (8.75, 25.00)	< 0.001

Patients were stratified according to mortality status. Continuous variables were assessed for normality using the Shapiro–Wilk test in the overall cohort and within each group. Normally distributed continuous variables were compared using the two-sample t-test, whereas non-normally distributed continuous variables were compared using the Wilcoxon rank-sum test. Categorical variables were compared using Pearson's chi-square test or Fisher's exact test, as appropriate. ALT, alanine aminotransferase; AST, aspartate aminotransferase; INR, international normalized ratio; CRP, C-reactive protein; IL-6, interleukin-6; AARC, Asian Pacific association for the study of the liver acute-on-chronic liver failure research consortium; MELD, Model For End-Stage Liver Disease; MELD-Na, sodium-adjusted Model For End-Stage Liver Disease; ALSS, artificial liver support system; SD, standard deviation; IQR, interquartile range.

### Baseline characteristics according to the median CALLY index

According to the median CALLY index of 34.51, 111 patients were classified into the low-CALLY group and 110 into the high-CALLY group. Patients with low CALLY were older and had higher rates of hepatic encephalopathy and renal failure (all *P* < 0.05). They also showed lower lymphocyte counts, hemoglobin, platelet counts, ALT, AST, and albumin levels, but higher INR, CRP, IL-6, Child–Pugh scores, MELD-Na scores, and more advanced AARC grades (all *P* < 0.05). Other baseline characteristics were comparable between the two groups (Table 2).

**Table 2:** Baseline characteristics according to the median CALLY index.

Characteristics	Low CALLY group (*N* = 111)	High CALLY group (*N* = 110)	*p*-value
Age (years), mean ± SD	51.09 ± 11.17	46.75 ± 10.75	0.004
Sex, *n* (%)			0.179
Female	33 (29.73)	24 (21.82)	
Male	78 (70.27)	86 (78.18)	
Hypertension history (Yes), *n* (%)	11 (9.91)	4 (3.64)	0.064
Diabetes history (Yes), *n* (%)	7 (6.31)	11 (10.00)	0.315
Cardiovascular disease history (Yes), *n* (%)	4 (3.60)	6 (5.45)	0.538
Hepatic encephalopathy (Yes), *n* (%)	32 (28.83)	19 (17.27)	0.041
Renal insufficiency (Yes), *n* (%)	15 (13.51)	0 (0.00)	< 0.001
White blood cell count ( × 10^9^/L), median (IQR)	5.48 (4.19, 8.38)	6.34 (4.22, 8.18)	0.553
Neutrophil count ( × 10^9^/L), median (IQR)	3.57 (2.54, 5.74)	3.74 (2.48, 4.89)	0.305
Lymphocyte count ( × 10^9^/L), median (IQR)	0.86 (0.59, 1.06)	1.45 (1.08, 1.91)	< 0.001
Hemoglobin (g/L), median (IQR)	118.00 (93.50, 137.50)	138.00 (122.00, 151.00)	< 0.001
Platelet count ( × 10^9^/L), median (IQR)	86.00 (63.50, 129.50)	124.00 (89.00, 171.50)	< 0.001
ALT (U/L), median (IQR)	105.60 (46.10, 349.05)	323.45 (115.58, 642.73)	< 0.001
AST (U/L), median (IQR)	151.60 (91.85, 318.25)	264.70 (137.58, 628.40)	< 0.001
Total bilirubin (μmol/L), median (IQR)	320.90 (235.46, 405.35)	286.20 (224.03, 377.77)	0.106
Albumin (g/L), median (IQR)	29.60 (26.40, 32.15)	32.21 (29.13, 36.48)	< 0.001
INR, median (IQR)	1.81 (1.61, 2.23)	1.68 (1.43, 2.09)	0.023
CRP (mg/L), median (IQR)	19.60 (14.70, 38.30)	6.46 (3.92, 8.77)	< 0.001
IL-6 (pg/ml), median (IQR)	18.00 (9.24, 53.75)	7.65 (3.91, 14.95)	< 0.001
AARC grade, *n* (%)			< 0.001
I	27 (24.32)	63 (57.27)	
II	62 (55.86)	39 (35.45)	
III	22 (19.82)	8 (7.27)	
Child-Pugh score, median (IQR)	11.00 (11.00, 12.50)	10.00 (9.00, 11.75)	< 0.001
MELD score, median (IQR)	19.42 (16.09, 23.39)	19.05 (16.47, 21.17)	0.356
MELD-Na score, median (IQR)	26.71 (21.42, 40.00)	20.46 (18.20, 24.87)	< 0.001

Patients were stratified according to the median CALLY index: low CALLY, ≤ 34.51; high CALLY, >34.51. Age was compared using Student's t-test; other continuous variables were compared using the Wilcoxon rank-sum test. Categorical variables were compared using Pearson's chi-square test or Fisher's exact test, as appropriate. CALLY, C-reactive protein–albumin–lymphocyte index; SD, standard deviation; IQR, interquartile range; ALT, alanine aminotransferase; AST, aspartate aminotransferase; INR, international normalized ratio; CRP, C-reactive protein; IL-6, interleukin-6; AARC, Asian Pacific Association for the Study of the Liver ACLF Research Consortium; MELD, Model for End-Stage Liver Disease; MELD-Na, sodium-adjusted Model for End-Stage Liver Disease.

### Association of CALLY with all-cause mortality

**Figure 2:**
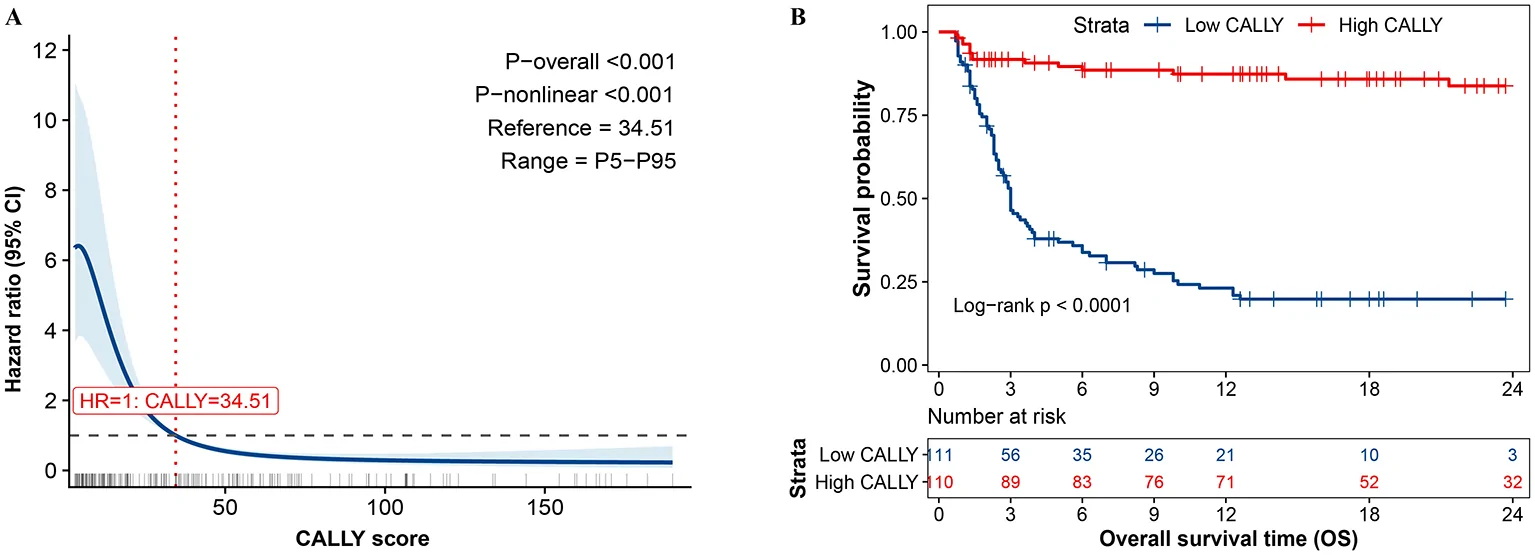
Association between baseline CALLY index and all-cause mortality. **(A)** Restricted cubic spline analysis showing the association between baseline CALLY score and the hazard ratio for all-cause mortality. The median CALLY value of 34.51 was used as the reference point, and the analysis was restricted to the 5th−95th percentile range. The solid line represents the estimated hazard ratio, and the shaded area represents the 95% confidence interval. **(B)** Kaplan–Meier survival curves according to CALLY groups. CALLY, C-reactive protein–albumin–lymphocyte index; HR, hazard ratio; CI, confidence interval.

Restricted cubic spline analysis showed a significant nonlinear inverse association between the baseline CALLY index and the risk of all-cause mortality. After restricting the analysis to the 5th−95th percentile range, the risk of all-cause mortality decreased markedly with increasing CALLY levels and tended to plateau at higher CALLY values (overall association, P < 0.001; nonlinearity, *P* < 0.001; Figure 2A). Kaplan–Meier survival analysis further demonstrated that patients in the high-CALLY group had significantly better overall survival than those in the low-CALLY group. The survival curves separated early during follow-up, and the difference persisted throughout the follow-up period (log-rank *P* < 0.0001; Figure 2B).

Cox proportional hazards regression analysis further confirmed the inverse association between the CALLY index and all-cause mortality. A higher CALLY index was significantly associated with a lower risk of all-cause mortality in the crude model, in Model 1 adjusted for age and sex, and in Model 2 further adjusted for histories of hypertension, diabetes mellitus, and cardiovascular disease. In Model 2, each 1-unit increase in CALLY was associated with an approximately 2.9% reduction in the risk of all-cause mortality (HR = 0.971, 95% CI: 0.962–0.980, *P* < 0.001). After log2 transformation, each doubling of the CALLY index was associated with a 42.0% reduction in mortality risk (HR = 0.580, 95% CI: 0.516–0.652, *P* < 0.001). When patients were categorized according to the median CALLY value of 34.51, those in the high-CALLY group had a significantly lower risk of death than those in the low-CALLY group (HR = 0.114, 95% CI: 0.065–0.201, *P* < 0.001). In the tertile-based analysis, both the middle and highest tertiles were associated with significantly lower mortality risks compared with the lowest tertile, and a significant decreasing trend in mortality risk was observed across increasing CALLY tertiles (*P* for trend < 0.001; Table 3). To assess the potential influence of liver transplantation on the results, a sensitivity analysis was further performed. Only one patient in the cohort underwent liver transplantation during follow-up. After censoring this patient at the time of transplantation, the inverse association between CALLY and all-cause mortality remained robust (Supplementary Table 1).

**Table 3:** Association of the CALLY index with the risk of all-cause mortality.

CALLY	Crude		Model1		Model2	
	HR (95%CI)	*P*	HR (95%CI)	*P*	HR (95%CI)	*P*
CALLY	0.970 (0.961, 0.979)	< 0.001	0.971 (0.962, 0.980)	< 0.001	0.971 (0.962, 0.980)	< 0.001
Log2 (CALLY)	0.587 (0.526, 0.654)	< 0.001	0.586 (0.523, 0.657)	< 0.001	0.580 (0.516, 0.652)	< 0.001
Binary groups						
G1 (ref)	–	–	–	–	–	–
G2	0.108 (0.062, 0.189)	< 0.001	0.116 (0.066, 0.204)	< 0.001	0.114 (0.065, 0.201)	< 0.001
Tertile groups						
T1 (ref, lowest tertile)	–	–	–	–	–	–
T2	0.210 (0.130, 0.339)	< 0.001	0.211 (0.129, 0.344)	< 0.001	0.212 (0.130, 0.346)	< 0.001
T3	0.070 (0.034, 0.142)	< 0.001	0.078 (0.038, 0.160)	< 0.001	0.078 (0.038, 0.161)	< 0.001
*P* for trend		< 0.001		< 0.001		< 0.001

HRs and 95% CIs were estimated using Cox proportional hazards regression models. Mortality was defined as all-cause death. For continuous CALLY, the HR represents the risk change per 1-unit increase; for log2(CALLY), the HR represents the risk change per doubling of the CALLY index. Binary groups were defined by the median CALLY value: G1, CALLY ≤ 34.510; G2, CALLY > 34.510. Tertile groups were defined according to the distribution of CALLY, with T1 as the lowest tertile and reference group, T2 as the middle tertile, and T3 as the highest tertile. P for trend was calculated by modeling tertiles as an ordinal variable. The crude model was unadjusted. Model 1 was adjusted for age and sex. Model 2 was further adjusted for hypertension history, diabetes history, and cardiovascular disease history. Two-sided P < 0.05 was considered statistically significant.

In addition, as a supplementary analysis, we assessed the cross-sectional associations between baseline CALLY and major baseline complications. After adjustment for age, sex, and histories of hypertension, diabetes mellitus, and cardiovascular disease, CALLY was not independently associated with hepatic encephalopathy, whereas a higher CALLY index was significantly associated with lower odds of baseline renal insufficiency (Supplementary Table 2).

### Predictive performance of CALLY

**Figure 3:**
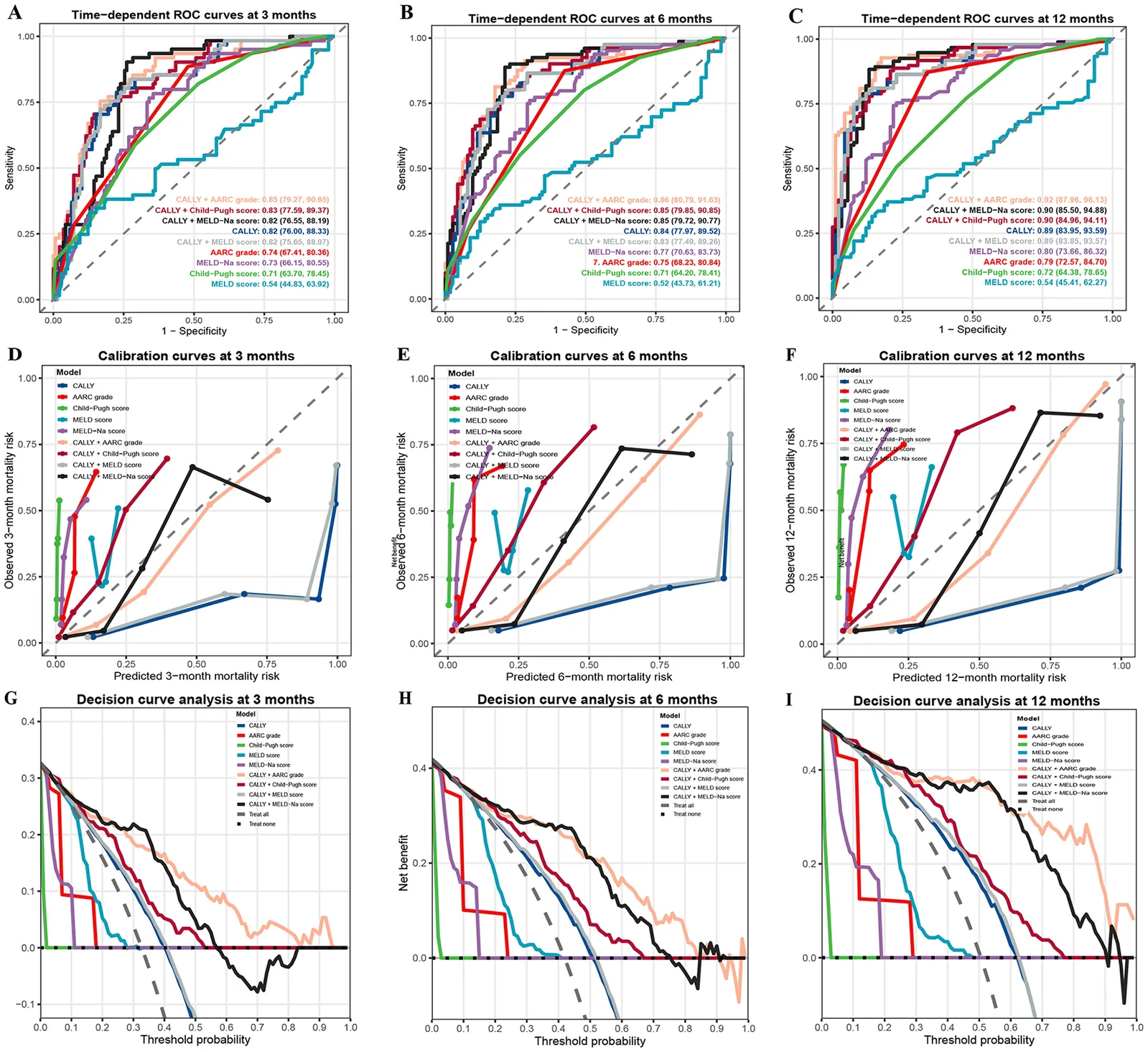
Predictive performance and clinical utility of CALLY for all-cause mortality. **(A–C)** Time-dependent ROC curves of CALLY, conventional prognostic scores, and CALLY-based combined models for predicting all-cause mortality at 3, 6, and 12 months, respectively. For ROC analyses, marker direction was specified so that higher risk scores corresponded to higher predicted mortality risk. For CALLY alone, inverse CALLY was used only to ensure the correct direction of prediction. **(D–F)** Calibration curves comparing predicted and observed mortality risks at 3, 6, and 12 months, respectively. **(G–I)** Decision curve analysis showing the net clinical benefit of CALLY alone and CALLY-based combined models at 3, 6, and 12 months, respectively.

To further evaluate the prognostic value of the baseline CALLY index for all-cause mortality in HBV-ACLF patients receiving ALSS therapy, time-dependent ROC curves were constructed at 3, 6, and 12 months. CALLY alone showed good discriminative ability for predicting all-cause mortality, with AUCs of 0.822 (95% CI: 0.760–0.883), 0.837 (95% CI: 0.780–0.895), and 0.888 (95% CI: 0.840–0.936) at 3, 6, and 12 months, respectively. At each follow-up time point, the AUC of CALLY was higher than those of AARC grade, Child–Pugh score, MELD score, and MELD-Na score, suggesting that baseline CALLY had stable predictive performance for short- and intermediate-term mortality risk (Figures 3A–C and Supplementary Table 3). Further assessment of the incremental predictive value of CALLY showed that, after adding CALLY to AARC grade, Child–Pugh score, MELD score, and MELD-Na score, the AUCs of the combined models were significantly higher than those of the corresponding reference models at 3, 6, and 12 months (Figures 3A–C and Supplementary Table 3).

Brier score analysis showed that, compared with the corresponding reference models, the addition of CALLY to AARC grade, Child–Pugh score, and MELD-Na score reduced the Brier scores at 3, 6, and 12 months, indicating improved overall predictive accuracy of these models (Supplementary Table 3). Calibration curves demonstrated that the combined models incorporating CALLY generally showed favorable calibration, with good agreement between predicted and observed risks (Figures 3D–F). Decision curve analysis further indicated that CALLY alone and the combined models integrating CALLY with conventional prognostic scores achieved higher net benefits across several clinically relevant threshold probability ranges. Among these models, CALLY combined with AARC grade and CALLY combined with MELD-Na score showed particularly favorable clinical net benefits (Figures 3G–I).

## Discussion

In this study, we systematically evaluated the clinical significance of the baseline CALLY index in patients with HBV-ACLF receiving ALSS therapy. We found that patients with low CALLY had more severe systemic inflammation, poorer nutritional and immune status, and more advanced disease severity at baseline. More importantly, baseline CALLY was significantly and inversely associated with all-cause mortality, and patients with high CALLY showed markedly better overall survival. Time-dependent ROC analyses further demonstrated that CALLY had good and stable predictive performance for mortality at 3, 6, and 12 months. In addition, adding CALLY to conventional prognostic scores improved model discrimination, calibration, and clinical utility.

The prognostic value of CALLY is biologically plausible because it integrates three clinically relevant domains in HBV-ACLF, namely inflammation, nutritional status, and immunity, through CRP, albumin, and lymphocyte count, respectively (12). These components are closely linked to the major pathophysiological processes driving disease progression.

First, systemic inflammation is a central feature of ACLF and an important determinant of outcome (12, 13). In HBV-ACLF, bacterial infection is a common precipitating event, while sterile inflammation caused by hepatocyte injury, HBV reactivation, and the release of damage-associated molecular patterns may further amplify inflammatory signaling (14–16). CRP, as a widely used acute-phase reactant, reflects inflammatory burden and has been associated with disease severity and prognosis in multiple clinical settings (17). Accordingly, lower CRP levels, corresponding to higher CALLY scores, may indicate a less pronounced inflammatory state and a more favorable prognosis.

Second, albumin reflects not only hepatic synthetic capacity and nutritional reserve, but also multiple protective biological functions, including antioxidant, anti-inflammatory, endothelial-stabilizing, and immunomodulatory effects (18). Hypoalbuminemia may therefore indicate impaired liver function, ongoing inflammatory consumption, and reduced physiological resilience (19). This provides a mechanistic basis for the association between lower CALLY and worse clinical outcomes.

Third, immune dysregulation is increasingly recognized as a hallmark of HBV-ACLF (20). Excessive immune activation may coexist with immune exhaustion or immune paralysis, particularly in the setting of intestinal barrier dysfunction and microbial translocation (4). Lymphocyte count is a simple and accessible indicator of immune status (21). In patients with HBV-ACLF, reduced lymphocyte count may reflect immune exhaustion and may predispose to secondary infection, organ failure, and death. Taken together, a low CALLY score may identify a high-risk phenotype characterized by concurrent inflammation, malnutrition, and immune dysfunction.

Our findings regarding complications further support the clinical relevance of CALLY. Systemic inflammation is a highly energy-consuming process that may disrupt peripheral organ homeostasis (12). In the present study, we focused on two representative complications, namely HE and renal dysfunction. Brain failure is an important form of organ failure in ACLF, which not only markedly increases mortality risk but also poses unique challenges in clinical management (22). Renal dysfunction occurs in approximately 30% to more than 95% of patients with ACLF, indicating major organ involvement and a close association with poor prognosis (23, 24).

Previous studies have suggested that the CALLY index has prognostic value in critically ill patients and infection-related conditions (25–28). Our study extends these findings to HBV-ACLF patients receiving ALSS therapy. As CALLY can be easily calculated from routine laboratory parameters before treatment, it may serve as a practical and low-cost tool for clinical risk stratification. Notably, CALLY provided incremental prognostic value beyond conventional scoring systems. CALLY alone showed good discriminative ability for predicting 3, 6, and 12-month all-cause mortality and showed higher AUCs than AARC grade, Child–Pugh score, MELD score, and MELD-Na score at each time point. Moreover, adding CALLY to these conventional scores significantly increased the AUCs of the corresponding models. Brier score analysis, calibration curves, and decision curve analysis further supported the improved predictive accuracy and clinical net benefit of CALLY-based combined models, particularly those combining CALLY with AARC grade or MELD-Na score. These findings indicate that CALLY may complement, rather than replace, existing prognostic scores and may help refine individualized risk assessment before ALSS therapy. Nevertheless, these predictive analyses should be interpreted as exploratory assessments of apparent model performance rather than evidence of a fully validated clinical prediction tool. The clinical applicability of CALLY for individualized risk stratification requires further confirmation through internal validation, external validation, and multicenter prospective studies.

### Limitations

Several limitations should be acknowledged. First, this was a single-center retrospective study and is therefore subject to selection bias and residual confounding. Second, only baseline CALLY was assessed, and the prognostic significance of its dynamic changes during ALSS treatment could not be evaluated. Third, ALSS modality and treatment frequency varied among patients and were determined according to disease severity, coagulation status, hemodynamic stability, and clinical response. Therefore, indication bias and residual confounding related to ALSS treatment cannot be fully excluded, particularly because the number of ALSS sessions was a post-baseline variable that may have been influenced by early treatment response and survival status. Fourth, in the present cohort, only one patient underwent liver transplantation during follow-up, a formal competing-risk analysis was not feasible. Although the association between CALLY and all-cause mortality remained consistent after censoring this patient at the time of transplantation, residual bias related to transplant eligibility, timing of transplantation, and clinical decision-making cannot be completely ruled out. Multicenter prospective studies are needed to validate our findings and to clarify the value of dynamic CALLY monitoring.

## Conclusion

In patients with HBV-ACLF receiving ALSS therapy, a low baseline CALLY index was associated with a higher risk of all-cause mortality and poorer overall survival. CALLY showed good predictive performance for short- and intermediate-term mortality and improved prognostic assessment when incorporated into conventional scoring systems. As a simple and readily available composite biomarker, CALLY may be useful for pre-ALSS risk stratification and individualized prognostic evaluation.

## Data Availability

The raw data supporting the conclusions of this article will be made available by the authors, without undue reservation.
