# Supplementary material for: Baseline CALLY index is associated with all-cause mortality in HBV-ACLF patients receiving ALSS therapy
Source: Front Med (Lausanne). 2026 Aug 21;13:1827310. doi: 10.3389/fmed.2026.1827310 (PMC13542649; doi:10.3389/fmed.2026.1827310)
Supplement: Supplementary file 1 [file Table_1.docx]

| Supplementary Table 1. Association of the CALLY index with the risk of all-cause mortality | | | | | | |
| --- | --- | --- | --- | --- | --- | --- |
|  | Crude | | Model1 | | Model2 | |
|  | HR(95%CI) | *P* | HR(95%CI) | *P* | HR(95%CI) | *P* |
| CALLY | 0.970 (0.961, 0.979) | <0.001 | 0.971 (0.962, 0.980) | <0.001 | 0.971 (0.962, 0.980) | <0.001 |
| Log2(CALLY) | 0.587 (0.527, 0.654) | <0.001 | 0.586 (0.523, 0.658) | <0.001 | 0.580 (0.516, 0.652) | <0.001 |
| Binary groups |  |  |  |  |  |  |
| G1 (ref) |  |  |  |  |  |  |
| G2 | 0.108 (0.062, 0.189) | <0.001 | 0.117 (0.066, 0.205) | <0.001 | 0.115 (0.065, 0.202) | <0.001 |
| Tertile groups |  |  |  |  |  |  |
| T1 (ref, lowest tertile) |  |  |  |  |  |  |
| T2 | 0.218 (0.136, 0.349) | <0.001 | 0.219 (0.135, 0.355) | <0.001 | 0.221 (0.136, 0.358) | <0.001 |
| T3 | 0.062 (0.030, 0.132) | <0.001 | 0.070 (0.033, 0.148) | <0.001 | 0.070 (0.033, 0.149) | <0.001 |
| P for trend |  | <0.001 |  | <0.001 |  | <0.001 |
| Note: This table presents the results of a sensitivity analysis in which one patient who underwent liver transplantation during follow-up was censored on the date of transplantation. HRs and 95% CIs were estimated using Cox proportional hazards regression models. Mortality was defined as all-cause death. For continuous CALLY, the HR represents the risk change per 1-unit increase; for log2(CALLY), the HR represents the risk change per doubling of the CALLY index. Binary groups were defined according to the median CALLY value: G1, CALLY ≤ 34.510; G2, CALLY > 34.510. Tertile groups were defined according to the distribution of CALLY, with T1 as the lowest tertile and reference group, T2 as the middle tertile, and T3 as the highest tertile. P for trend was calculated by modeling tertiles as an ordinal variable. The crude model was unadjusted. Model 1 was adjusted for age and sex. Model 2 was further adjusted for hypertension history, diabetes history, and cardiovascular disease history. Two-sided P < 0.05 was considered statistically significant. | | | | | | |
