# Supplementary material for: Baseline CALLY index is associated with all-cause mortality in HBV-ACLF patients receiving ALSS therapy
Source: Front Med (Lausanne). 2026 Aug 21;13:1827310. doi: 10.3389/fmed.2026.1827310 (PMC13542649; doi:10.3389/fmed.2026.1827310)
Supplement: Supplementary file 2 [file Table_2.docx]

| Supplementary Table 2. Cross-sectional logistic regression analyses of complications according to CALLY index | | | | |
| --- | --- | --- | --- | --- |
| **Complication** | **Univariable analysis** | | **Multivariable analysis** | |
|  | OR (95% CI) | *P* value | aOR (95% CI) | *P* value |
| Hepatic encephalopathy | 0.997 (0.993–1.002) | 0.201 | 0.998 (0.993–1.002) | 0.295 |
| Renal insufficiency | 0.917 (0.862–0.976) | 0.006 | 0.912 (0.854–0.975) | 0.007 |
| Multivariable model adjusted for age, sex, hypertension, diabetes, and cardiovascular disease. ORs are expressed per 1-unit increase in CALLY index. | | | | |
