# Supplementary material for: Baseline CALLY index is associated with all-cause mortality in HBV-ACLF patients receiving ALSS therapy
Source: Front Med (Lausanne). 2026 Aug 21;13:1827310. doi: 10.3389/fmed.2026.1827310 (PMC13542649; doi:10.3389/fmed.2026.1827310)
Supplement: Supplementary file 3 [file Table_3.docx]

| **Supplementary Table 3. Predictive performance and incremental value of CALLY for all-cause mortality at 3, 6, and 12 months** | | | | | | | |
| --- | --- | --- | --- | --- | --- | --- | --- |
| **Follow-up time (months)** | **Model** | **Reference model** | **AUC (95% CI)** | **Brier score** | **Delta AUC** | ***P* value** | **Brier improvement** |
| 3 | CALLY | NA | 0.822 (0.760-0.883) | 0.425 | NA | NA | NA |
| 3 | AARC grade | NA | 0.739 (0.674-0.804) | 0.282 | NA | NA | NA |
| 3 | Child-Pugh score | NA | 0.711 (0.637-0.784) | 0.335 | NA | NA | NA |
| 3 | MELD score | NA | 0.544 (0.448-0.639) | 0.255 | NA | NA | NA |
| 3 | MELD-Na score | NA | 0.734 (0.662-0.805) | 0.302 | NA | NA | NA |
| 3 | CALLY + AARC grade | AARC grade | 0.850 (0.793-0.907) | 0.156 | 0.111 | <0.001 | 0.127 |
| 3 | CALLY + Child-Pugh score | Child-Pugh score | 0.835 (0.776-0.894) | 0.198 | 0.124 | <0.001 | 0.137 |
| 3 | CALLY + MELD score | MELD score | 0.819 (0.757-0.881) | 0.392 | 0.275 | <0.001 | -0.137 |
| 3 | CALLY + MELD-Na score | MELD-Na score | 0.824 (0.765-0.882) | 0.177 | 0.090 | 0.005 | 0.125 |
| 6 | CALLY | NA | 0.837 (0.780-0.895) | 0.416 | NA | NA | NA |
| 6 | AARC grade | NA | 0.745 (0.682-0.808) | 0.354 | NA | NA | NA |
| 6 | Child-Pugh score | NA | 0.713 (0.642-0.784) | 0.439 | NA | NA | NA |
| 6 | MELD score | NA | 0.525 (0.437-0.612) | 0.305 | NA | NA | NA |
| 6 | MELD-Na score | NA | 0.772 (0.706-0.837) | 0.378 | NA | NA | NA |
| 6 | CALLY + AARC grade | AARC grade | 0.862 (0.808-0.916) | 0.170 | 0.117 | <0.001 | 0.184 |
| 6 | CALLY + Child-Pugh score | Child-Pugh score | 0.854 (0.798-0.908) | 0.215 | 0.140 | <0.001 | 0.224 |
| 6 | CALLY + MELD score | MELD score | 0.834 (0.775-0.893) | 0.389 | 0.309 | <0.001 | -0.084 |
| 6 | CALLY + MELD-Na score | MELD-Na score | 0.853 (0.797-0.908) | 0.178 | 0.081 | 0.011 | 0.200 |
| 12 | CALLY | NA | 0.888 (0.840-0.936) | 0.390 | NA | NA | NA |
| 12 | AARC grade | NA | 0.786 (0.726-0.847) | 0.406 | NA | NA | NA |
| 12 | Child-Pugh score | NA | 0.715 (0.644-0.787) | 0.532 | NA | NA | NA |
| 12 | MELD score | NA | 0.538 (0.454-0.623) | 0.339 | NA | NA | NA |
| 12 | MELD-Na score | NA | 0.800 (0.737-0.863) | 0.441 | NA | NA | NA |
| 12 | CALLY + AARC grade | AARC grade | 0.920 (0.880-0.961) | 0.141 | 0.134 | <0.001 | 0.265 |
| 12 | CALLY + Child-Pugh score | Child-Pugh score | 0.895 (0.850-0.941) | 0.210 | 0.180 | <0.001 | 0.321 |
| 12 | CALLY + MELD score | MELD score | 0.887 (0.838-0.936) | 0.367 | 0.349 | <0.001 | -0.029 |
| 12 | CALLY + MELD-Na score | MELD-Na score | 0.902 (0.855-0.949) | 0.157 | 0.102 | 0.001 | 0.284 |
| **Note.** AUC, area under the curve; CI, confidence interval; MELD, Model for End-Stage Liver Disease; MELD-Na, sodium-adjusted Model for End-Stage Liver Disease; NA, not applicable. Brier score evaluates overall prediction error, with lower values indicating better accuracy. Delta AUC was calculated as the AUC of the combined model minus the AUC of the corresponding reference model. The P value compares the AUC of each combined model with its reference model. Brier improvement was calculated as the Brier score of the reference model minus the Brier score of the combined model; positive values indicate lower prediction error after adding CALLY. Original CI limits were stored as percentages and were converted to the 0-1 scale. | | | | | | | |
